# Increased frequency of Th17 cells and IL-17 levels are associated with low bone mineral density in postmenopausal women

**DOI:** 10.1038/s41598-021-95640-0

**Published:** 2021-08-09

**Authors:** Hetal Bhadricha, Vainav Patel, Amit Kumar Singh, Lalita Savardekar, Anushree Patil, Suchitra Surve, Meena Desai

**Affiliations:** 1 Molecular Immunodiagnostics Division, ICMR-National Institute for Research in Reproductive Health, Mumbai, India; 2 Department of Biochemistry and Virology, ICMR-National Institute for Research in Reproductive Health, Mumbai, India; 3 Department of Clinical Research, ICMR-National Institute for Research in Reproductive Health, Mumbai, India

**Keywords:** Cytokines, Inflammation, Osteoimmunology

## Abstract

Osteoporosis is one of the chronic and often neglected bone diseases in aging postmenopausal women that affect the quality of life. Studies on ovariectomized mice models indicated the reciprocal role of Th17 cells and Treg cells in the aetiology of osteoporosis. While Th17 cells promote osteoclastogenesis, Treg cells exhibit anti-osteoclastogenic activity. This exploratory study aimed to determine the difference in the frequency of these T-cell subtypes in pre-and postmenopausal women and to examine their association with BMD. In our study, the frequency of Treg cells, analyzed by flow cytometry, did not differ between pre-and postmenopausal women. However, plasma levels of IL-10 along with IL-10^+^CD4^+^T cells were higher in post- compared to premenopausal women. The frequency of Th17 cells was higher in postmenopausal women irrespective of their BMD, however, only postmenopausal women with low BMD had elevated IL-17 levels and their T-scores were associated with Th17 frequency. Collectively, the results suggest that estrogen insufficiency in postmenopausal women may lead to increased Th17 cell frequency and elevated IL-17 levels which are associated with low BMD. This study highlights, Th17 cells and IL-17 as key players in the pathogenesis of osteoporosis and they can be the potential targets for immunotherapy in the treatment of osteoporosis.

## Introduction

Osteoporosis, a chronic condition characterized by low bone mineral density (BMD) and fragile skeletal tissue predisposes individuals to an increased risk of fractures^1^. Genetic, endocrine, environmental, lifestyle factors and curtailed peak bone mass are some of the known causal factors for the observed low bone mass. In addition to these factors, growing evidence suggests that the activated immune profile also plays a potentially critical role both in the normal bone remodeling process and in the etiology of osteoporosis^2–4^. Various studies have suggested the significant role of the immune system in bone remodeling however, the exact mechanism by which it affects osteoclastogenesis is yet to be fully elucidated. This led to the emergence of a modern field, osteoimmunology, to explore the role of immune cells and immune-derived factors like cytokines in the occurrence of osteoporosis^5–7^. T helper cells along with other immune cells and cytokines are involved in bone homeostasis. Although several studies have reported the role of inflammatory markers in the pathology of osteoporosis, only a few studies have elucidated the role of T cells in the pathophysiology of osteoporosis. Of late, a new term has been proposed by the Srivastava group as “immunoporosis: the immunology of osteoporosis”, a novel field with special emphasis on the role of various T cell subsets and osteoporosis^8,9^. Among the immune factors, primary drivers of bone remodeling are T helper 17 (Th17) cells and regulatory T cells (Tregs) that have opposing actions in maintaining bone homeostasis, especially osteoclastogenesis^10,11^.

Tregs are anti-inflammatory cells that maintain peripheral tolerance by suppressing and regulating the activity of inflammatory T cells. Several groups have demonstrated inhibitory effects of Treg cells on osteoclastogenesis through the secretion of cytokines like TGF-β and IL-10^12,13^, however, there is still no consensus regarding the exact inhibitory mechanisms^14–16^. In addition, it was demonstrated that the inhibitory effect of Treg cells on osteoclastogenesis was enhanced in presence of estrogen^12,17^, thus suggesting its involvement in estrogen-mediated regulation of bone metabolism and its protective function in bone.

Th17, an inflammatory effector subset, on the other hand, induces osteoclastogenesis and enhances bone loss under estrogen-deficient conditions. Studies on ovariectomized (OVX) mice demonstrated a rise in Th17 differentiation and subsequent increase in Th17 related cytokines like IL-17, TNF-α and IL-6 that potentially induce osteoclastogenesis^18–22^. Besides, it has been shown that the number of osteoclasts increases in presence of exogenous IL-17^23^. Further, some reports have demonstrated that treatment with anti-IL-17 antibody or silencing of IL-17 signaling prevented bone loss in OVX mice compared to wild-type mice thus reiterating the role of Th17 in increased osteoclastogenesis that contributes to the pathogenesis of osteoporosis^19,24,25^.

Interestingly, Th17 and Treg cells exhibit a great magnitude of plasticity, thus, these cells can switch to one another cell fate, change their function and phenotype according to the cytokine milieu that they encounter^26^. Since these cells are associated with each other, maintaining the balance between these cells becomes essential in preventing inflammatory disorder risk, including osteoporosis. Tipping of balance towards inflammatory Th17 cells may lead to increased osteoclastogenesis and promote bone resorption.

*In-vitro* and *in-vivo* studies strongly indicate an opposite and crucial role of both Th17 and Treg subsets in the pathogenesis of osteoporosis while human studies are lacking to substantiate their role in low bone mass. So far human studies have shown the association of estrogen deficiency with the expansion of T cells expressing TNF-α and RANKL^27,28^. Besides, other studies have majorly focused on the circulating levels and their association with BMD in healthy, osteopenic, and osteoporotic women^29,30^.

Human studies reporting the distribution of these cells and related immune factors that contribute to low BMD in postmenopausal women are not available to date. Here, we hypothesize that an imbalance in Th17/Treg results in low BMD conditions in postmenopausal women. Thus, the aim of the study was to analyze the differences in the proportion of circulatory Th17 and Treg cells in premenopausal and postmenopausal women. We also investigated the association of these cells with T-scores. The outcome of the present study may aid in understanding the role of the T cell subsets in low BMD conditions and studying alterations in these subsets can be valuable for the diagnosis and management of osteoporosis.

## Results

### Basic clinical characteristics of the study cohort

The clinical characteristics of the participants are illustrated in Table 1. Mean age and years since menopause was significantly high in Post-L group compared to Post-N, while, BMI differed between Pre-N and Post-N. T-scores and BMD at spine and femoral neck were significantly different between Pre-N and Pos-L (Supplementary Fig. 1), whereas, they were comparable between Pre-N and Post-N. On comparing biochemical parameters, we observed that vitamin D levels were significantly high in Post-L group compared to Pre-N, as these women were on vitamin D supplements, which was not recorded during enrolment. Both Post-L and Post-N had significantly lower levels of estradiol and higher CRP compared to the Pre-N.

**Table 1:** Baseline demographic and clinical characteristics of the study cohort (n = 71).

Parameters	Pre-N^**a**^ (n = 25)	Post-N^**b**^ (n = 21)	Post-L^**c**^ (n = 25)	*p*-value
Age (years)	33.0 ± 4.10	53.48 ± 3.96	57.60 ± 4.44	**a vs b < 0.0001** **a vs c < 0.0001** **b vs c 0.0019**
BMI (kg/m^2^)	24.41 ± 4.3	27.49 ± 3.97	25.08 ± 4.75	**a vs b 0.008** a vs c 0.462 b vs c 0.072
YSM	–	4.864 ± 3.028	10.92 ± 5.461	**b vs c < 0.0001**
BMD at spine (g/cm^2^)	1.196 ± 0.07	1.122 ± 0.08	0.721 ± 0.09	a vs b 0.107 **a vs c < 0.0001** **b vs c < 0.0001**
BMD at femoral neck (g/cm^2^)	1.01 ± 0.07	0.963 ± 0.08	0.672 ± 0.13	a vs b 0.081 **a vs c < 0.0001** **b vs c < 0.0001**
*T-scores* spine	0.05 ± 0.53	− 0.19 ± 0.79	− 3.87 ± 0.69	a vs b 0.308 **a vs c < 0.0001** **b vs c < 0.0001**
*T-scores* femoral neck	− 0.25 ± 0.56	− 0.44 ± 0.64	− 2.82 ± 0.36	a vs b 0.098 **a vs c < 0.0001** **b vs c < 0.0001**
** Biochemical and Hormonal Estimations**				
Calcium (mg/dL)	8.929 ± 0.32	9.358 ± 0.32	8.529 ± 0.28	**a vs b 0.019** a vs c 0.143 b vs c 0.311
Phosphorous (mg/dL)	5.013 ± 0.63	5.658 ± 0.66	5.115 ± 0.83	a vs b 0.069 a vs c 0.657 b vs c 0.071
CRP (mg/L)	2.898 ± 2.72	5.395 ± 4.82	4.748 ± 3.88	**a vs b 0.022** **a vs c 0.042** b vs c 0.877
Vitamin D (ng/mL)	20.36 ± 14.82	28.25 ± 16.77	31.02 ± 25.21	a vs b 0.061 **a vs c 0.045** b vs c 0.733
Estradiol (pg/mL)	167.4 ± 181.9	91.71 ± 31.37	101.41 ± 90.01	**a vs b 0.003** **a vs c 0.0006** b vs c 0.642

Values are represented as Mean ± SD; *p* value < 0.05 is considered significant and marked in bold.

Abbreviations: *Pre-N* premenopausal women with normal BMD, *Post-N* postmenopausal women with normal BMD, *Post-L* postmenopausal women with low BMD, *BMI* Body Mass Index, *YSM* Years since Menopause *BMD* Bone Mineral Density, *CRP* C-Reactive Protein.

### No changes in Treg cell frequency but decreased per cell expression of CD25 observed on Treg cells in postmenopausal women

**Figure 1:**
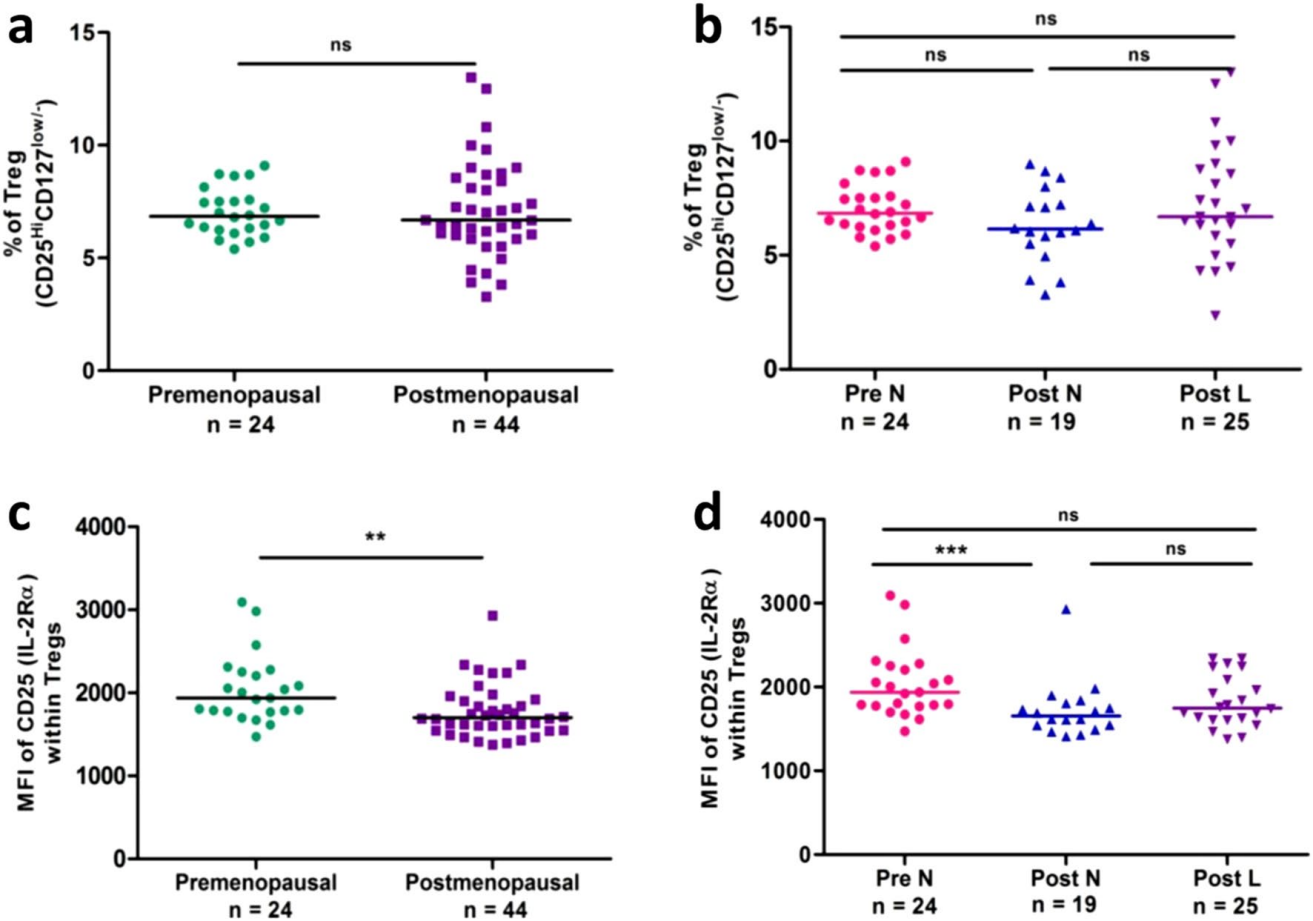
Frequencies of Treg cells and per-cell expression of CD25 on Treg cells in premenopausal women (n = 24) and postmenopausal women (n = 44) group (**a** & **c**) and in 3 cohorts (**b** & **d**): Pre-N (n = 24), Post-N (n = 19) and Post-L (n = 25). Data are expressed as scatter plots. Pairwise comparison (left panel) was done using the Mann–Whitney U test and multiple group comparison was done using the Kruskal–Wallis test with Dunn’s post-hoc analysis. The level of significance was considered only when *p* values were < 0.05. Abbreviations: *Pre-N* premenopausal women with normal BMD, *Post-N* postmenopausal women with normal BMD, *Post-L* postmenopausal women with low BMD, Bone Mineral density *BMD, Treg* regulatory T cells*, MFI* mean fluorescent intensity.

The gating strategy used for Treg cells is based on the expression of CD25 (IL-2Rα) and CD127 (IL-7Rα) on CD4^+^ T cells^31–33^. In this study, we have identified Treg cells as CD3^+^CD4^+^CD25^hi^CD127^low/−^ (Supplementary Fig 2, Fig. 3). Overall, the frequency of Treg cells between premenopausal and postmenopausal women was comparable (Fig. 1a). Further on we checked its frequency between Pre-N, Post-L and Post-N groups, but the differences were not significant amongst these groups (Fig. 1b).

IL-2 and IL-7 are critical regulators that function through their receptors CD25 (IL-2Rα) and CD127 (IL-7Rα) respectively for survival and proliferation of Treg cells. Hence, we also compared the Median fluorescent intensity (MFI) of CD25 per cell within Treg subset. Changes in MFI could represent a functional state for that particular marker. All postmenopausal women showed a significantly lower MFI of CD25 compared to premenopausal women (*p* = 0.0072) (Fig. 1c). The Post-N group showed a significant reduction in MFI (*p* = 0.004) compared to premenopausal women. Although a declining trend was observed in Post-L compared to Pre-N, the difference did not reach the level of significance (Fig. 1d). Moreover, women in both Post-L and Post-N groups had comparable levels of MFI of CD25 on Treg cells (Fig. 1d).

### Elevated frequencies of Th17 cells and IL-10^+^CD4^+^ T cells in postmenopausal women with low BMD

**Figure 2:**
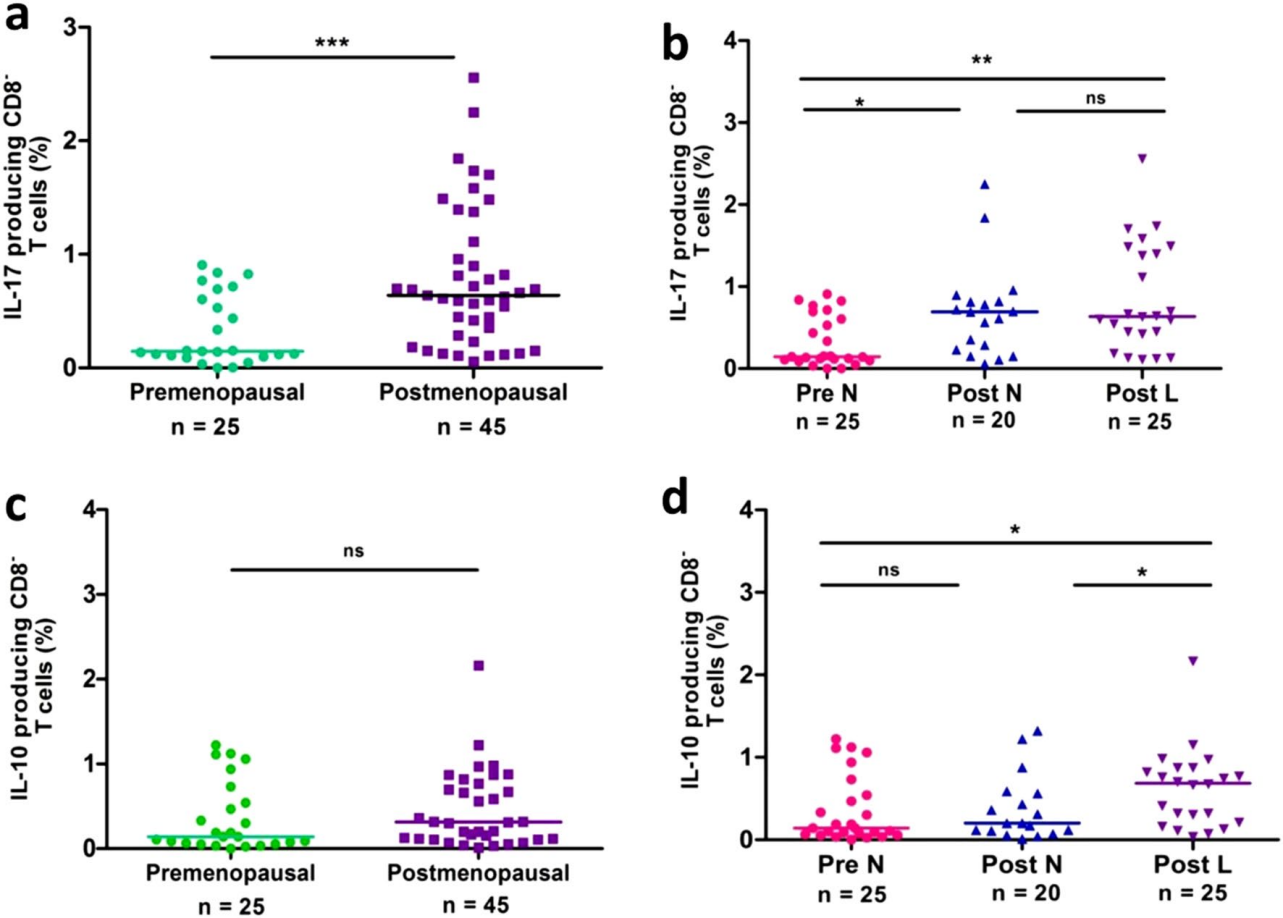
Frequencies of circulating Th17 (IL17^+^CD8^−^) cells and intracellular IL-10 levels by CD8^−^ T cells in premenopausal women (n = 25) and postmenopausal women (n = 45) (**a** & **c**) and in 3 cohorts (**b** & **d**): Pre-N (n = 25), Post-N (n = 20) and Post-L (n = 25). Data are expressed as scatter plots. Statistical significance was estimated by Kruskal–Wallis ANOVA with Dunn’s post-hoc analysis for multiple groups and Mann–Whitney U test for pairwise comparison. The level of significance was considered only when *p* values were < 0.05.

The functionality of Th17 and Treg cells depends on their capacity to produce IL-17 and IL-10 respectively. Therefore, we compared and quantified the percentage of Th17 and IL-10^+^CD4^+^ T (CD3^+^CD8^−^IL17^+^IL-10^+^) cells in freshly isolated PBMCs from women with varying BMD. Since PMA-ionomycin stimulation dramatically downregulates the surface expression of CD4, we applied a gating strategy of CD3^+^CD8^−^ to represent CD4^+^ T cells (Supplementary Fig. 4a). As shown in Fig. 2a, the frequencies of Th17 cells were significantly higher in postmenopausal women (*p* = 0.0009) than that of premenopausal women. Distinctly elevated Th17 frequency was observed in Post-L (*p* = 0.002) and Post-N (*p* = 0.012) when compared with those in Pre-N (Fig. 2b). However, there was no obvious difference between Post-N and Post-L groups (*p* =  > 0.05).

We next quantified the intracellular IL-10 production by CD4^+^ T cells. Though postmenopausal women had a higher frequency of IL-10^+^CD4^+^ T cells than premenopausal women, the difference was not statistically significant (Fig. 2c). Further on comparing Post-N, Post-L and Pre-N (Fig. 2d), the frequency of IL-10^+^CD4^+^ T cells was significantly elevated in Post-L when compared with Post-N (*p* = 0.042) and Pre-N (*p* = 0.014) (Fig. 2d). However, Pre-N and Post-N showed a comparable frequency of IL-10 producing CD4^+^ T cells.

### Altered Th17/Treg balance in postmenopausal women

**Figure 3:**
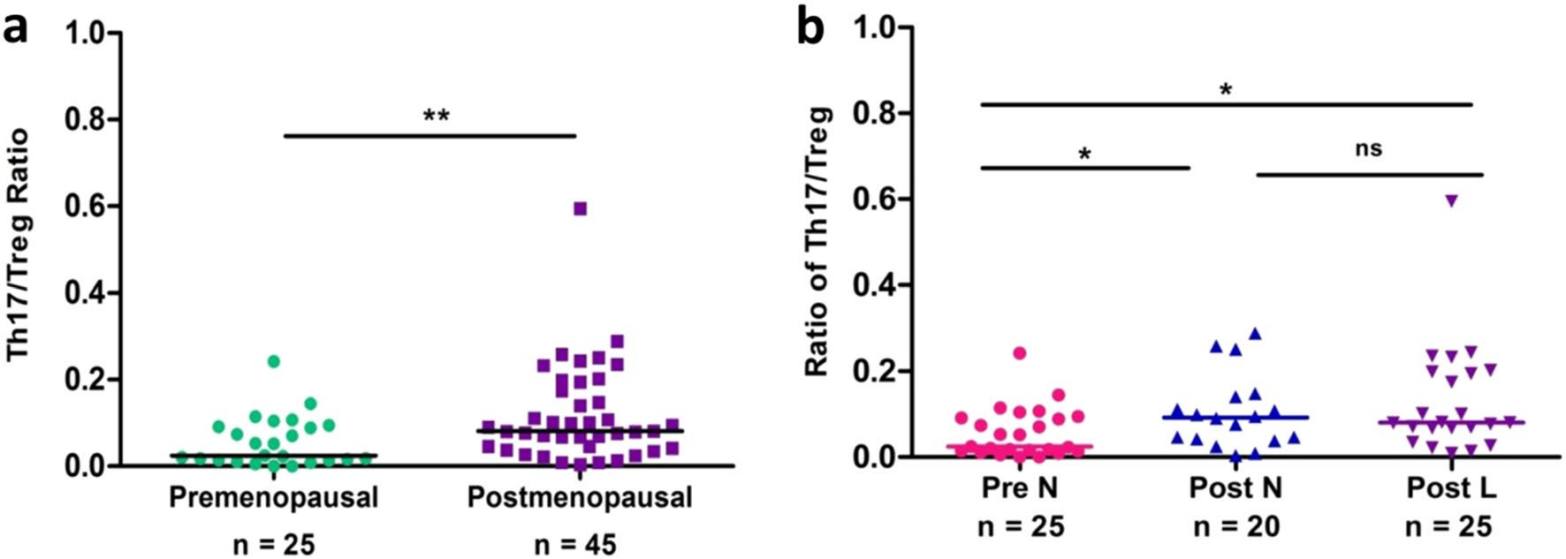
Ratio of circulating Th17/Treg cells in premenopausal women (n = 25) and postmenopausal women (n = 45) (**a**) and in 3 cohorts (**b**): Pre-N (n = 25), Post-N (n = 20) and Post-L (n = 25). Th17 cells were identified as CD3^+^CD8^−^1L-17^+^ and Treg as CD4^+^CD25^hi^CD127^low/−^. Statistical significance was estimated by Mann–Whitney U test for pairwise comparison and Kruskal–Wallis ANOVA with Dunn’s post-hoc analysis for multiple groups. The level of significance was considered only when *p* values were < 0.05.

The relation between Th17 cells and Treg cells was further investigated by comparing the Th17/Treg ratio in premenopausal and postmenopausal women. The Th17/Treg of postmenopausal women *(p* = 0.0065*)* was higher than that of premenopausal women (Fig. 3a). Moreover, this increase was observed in both Post-L (*p* = 0.0118) and Post-N (*p* = 0.039) when compared to those in the Pre-N group (Fig. 3b), while no statistical difference was observed between Post-N and Post-L groups.

### Plasma cytokines concentration in women with varying BMD

Differentiation of Treg and Th17 cells are reciprocally regulated by a common inducer, TGF-β. However, IL-6 together with TGF-β promotes the initial induction of Th17 cells reflecting that the cytokine environment governs the differentiation of either of the two subtypes^34,35^. We, thus examined, T helper cell-related cytokines in plasma of all the women using Luminex immunoassay. As shown in Table 2, the Post-L group had higher levels of IL-17 and IL-10 (*p* =  < 0.05), compared to Pre-N group, while, Post-N showed elevated levels of TNF-α and IL-10. On comparing low and normal BMD groups of postmenopausal women, we observed no difference in the levels of any of the cytokines (Table 2). Also, the levels of other cytokines such as TNF-α, IFN-γ, IL-7 and IL-4 did not differ between Post-N and Post-L groups (Supplementary Table 1). The levels of IL-7 and IFN-γ were elevated in Post-N and Post-L groups respectively with a concomitant increase in anti-inflammatory cytokine IL-4 in both the groups compared to Pre-N (Supplementary Table 1).

**Table 2:** Comparison of plasma level of cytokines between premenopausal and postmenopausal women with normal and low BMD.

Cytokines (pg/ml)	Pre-N (n = 21)	Post-N (n = 20 )	Post-L (n = 20)
IL-17	12.73 ± 8.63	19.08 ± 11.55	21.82 ± 14.17*
IL-6	8.06 ± 5.70	11.31 ± 6.85	9.78 ± 4.90
TNF-α	10.92 ± 5.29	19.69 ± 21.85*	11.16 ± 7.87
IL-10	0.58 ± 0.31	1.07 ± 0.69*	0.99 ± 0.61*
TGF-β	19.44 ± 11.13	22.39 ± 13.76	18.75 ± 9.38

Values are expressed as Mean ± SD. *p < 0.05; when compared to Pre-N.

Statistical significance was estimated using the Mann–Whitney U test.

Abbreviations: *Pre-N* premenopausal women with normal BMD, *Post-N* postmenopausal women with normal BMD, *Post-L* postmenopausal women with low BMD, *IL-17* Interleukin-17, IL-6 Interleukin-6, *TNF-α* Tumor Necrosis Factor-α, *IL-10* Interleukin-10, *TGF-β* Transforming growth factor-β.

### Association of CD4^+^ T cell subsets with T-scores at lumbar spine and femoral neck

As shown in Table 3, the frequencies of circulating Th17 cells and Th17/Treg ratio in the Post-L group negatively correlated with T-scores at both the sites (spine and femoral neck). However, frequencies of Treg cells did not correlate with T-scores. Further, Pre-N and Post-N did not show the association with any of the subsets with T-scores.

**Table 3:** Association of CD4^+^ T cell subsets with T score at lumbar spine and femoral neck in  the study cohort.

	Pre-N		Post-N		Post-L	
	r	*p*	r	*p*	r	*p*
**Lumbar Spine**						
% Th17 cells	0.312	0.121	0.180	0.448	**− 0.482**	**0.036**
% Treg cells	− 0.139	0.508	− 0.019	0.937	− 0.033	0.894
Th17/Treg	0.359	0.078	0.216	0.374	**− 0.561**	**0.013**
**Femoral neck**						
% Th17 cells	0.061	0.766	0.409	0.074	**− 0.469**	**0.021**
% Treg cells	0.128	0.542	0.012	0.961	− 0.071	0.743
Th17/Treg	0.024	0.908	0.325	0.175	**− 0.416**	**0.043**

Data are represented as Spearman’s rank correlation and level of significance. *p* value < 0.05 is considered significant and marked in bold.

Abbreviations: *Pre-N* premenopausal women with normal BMD, *Post-N* postmenopausal women with normal BMD, *Post-L* postmenopausal women with low BMD, *Th17* T helper 17, *Treg* regulatory T cells.

## Discussion

Osteoporosis is more prevalent in postmenopausal women as bone loss is accelerated due to declining levels of estrogen. Estrogen not only promotes osteoblast activity but also prevents bone resorption by osteoclasts. Apart from regulating the osteoblasts and osteoclasts, estrogen has an impact on immune cells too^36^. Estrogen is known to modulate the development and functions of CD4^+^ T cells^37^, however, the role of T cells in the pathogenesis of osteoporosis is not very clear.

In the present study, we hypothesized that a decline in estrogen level at menopause may lead to an imbalance in Th17/Treg cells which could supplement the inflammation in the bone milieu, leading to low BMD in postmenopausal women. Thus, we investigated the differences in the frequencies of Th17, Treg cells, and their ratios (Th17/Treg) between postmenopausal women and premenopausal women. Though few studies have reported that estrogen insufficiency is associated with an expansion of T cells expressing TNF-α^13,15^, no human study has investigated the association of CD4^+^ T cells with low BMD conditions. To the best of our knowledge, in this study, we reported the frequencies of Th17 and Treg cells in women and studied their association with BMD for the first time.

Th17 cells have been implicated in the pathogenesis of several inflammatory diseases including postmenopausal osteoporosis. In OVX mice, higher frequency of Th17 cells cause increased osteoclastogenesis by secreting IL-17, which in turn induces macrophages to secrete other inflammatory cytokines like IL-6, IL-1 and TNF-α. It is well established that inflammatory cytokines affect the bone remodeling process resulting in low bone mass. In our study, not only Th17 cells but also inflammatory cytokine IL-17 were significantly elevated in postmenopausal women with low BMD. Despite a very limited number of human studies^38,39^, the elevated frequency of Th17 in postmenopausal women with estrogen insufficiency is in agreement with the *in-vitro* and animal studies ^19,40^ who reported increased Th17 frequency and increased IL-17 cytokine levels in OVX induced estrogen-deficient mice^20–22^and these effects were reversed when treated with estrogen. Our observation preliminarily validates the outcomes of animal studies on humans.

Interestingly, postmenopausal women with normal BMD too had elevated levels of Th17 cells in circulation compared to premenopausal women. Similar to our findings, few studies have reported higher Th17 cells and IL-17A mRNA in healthy elderly individuals compared to younger subjects^41,42^. Inflammaging and years since menopause could be attributed to this increase in the frequency of Th17 cells^43,44^.

Treg (CD4^+^IL10^+^FOXP3^−^) cells have an immunosuppressive function and can inhibit bone resorption either by an inhibitory cytokine-dependent mechanism through IL-10 and TGF-β secretion or by cell contact-dependent mechanism via Cytotoxic T Lymphocyte-associated antigen-4 (CTLA4)^16–19^. In the present study, Treg cell frequency did not differ between premenopausal and postmenopausal women, also it did not vary with respect to BMD status in postmenopausal women. There has been no consensus on Treg frequency as few studies have reported a lower Treg frequency and reduced suppressive effect of Treg cells on osteoclast differentiation under estrogen insufficient condition^16,45^, whereas, few others reported an increase in Treg cell function in postmenopausal women due to the aging^46–48^. Another reason could be the high mean levels of vitamin D in osteoporotic women. Vitamin D is known to promote differentiation of Treg and also increases the level of anti-inflammatory cytokines like IL-10 ^49^. Further, elevated plasma levels of IL-10 along with a higher frequency of IL-10^+^CD4^+^T cells was seen in postmenopausal women, irrespective of their BMD status, compared to premenopausal women. Similar to our observation, Vural et al.have reported a rise in plasma IL-10 along with other anti-inflammatory cytokines in postmenopausal women and considered it to be a compensatory mechanism to counteract proinflammatory TNFα, thus balancing its pro-osteoclastogenic and oxidative stress-inducing effects that take place during menopause^50^.

Another interesting observation was that there was a reduction in per-cell expression of CD25 in postmenopausal women compared to premenopausal women, even though the Treg cell frequencies did not vary between these groups. This was an expected finding as reports have suggested that CD4^+^ T cells with higher CD25 expression have a greater suppressive activity^51,52^. A recent study has also demonstrated increased expression of CD25 in presence of estrogen in healthy females^53^. This suggests that estrogen regulates the expression of CD25 and thereby affects the suppressive activity of Treg cells, while it is important to note that we have not been able to demonstrate the function.

Overall, Th17/Treg was higher in postmenopausal women with normal as well as low BMD compared to premenopausal women. It is a well-established fact that estrogen can stimulate proliferation and differentiation of Treg cells^17^ and inhibit differentiation of Th17 cells^54^. Thus, the shift in Th17/Treg paradigm that we observe from premenopause to postmenopause can be attributed to estrogen insufficiency in the latter stage. Moreover, we observed a negative correlation of Th17 frequency and Th17/Treg with T-scores at the spine and femoral neck in postmenopausal women with low BMD. This further strengthens our hypothesis that estrogen insufficiency leads to a circulatory imbalance in Th17/Treg, which may augment the micro-inflammation and resorption in the bone milieu, resulting in excessive bone loss in postmenopausal women.

To ascertain the role of Th17 cells and Treg cells in the pathogenesis of osteoporosis, our approach was limited to determine the differences in frequencies of these subtypes between premenopausal women and postmenopausal women and study their association with BMD. Yet, our preliminary data provide evidence that links pro-inflammatory Th17 cells and cytokines to bone loss in humans. Since our study comprised healthy postmenopausal women, many other factors responsible for inflammation could not be studied. The other limitation of our study was that few postmenopausal women were on vitamin D supplementation. Although the supplementation was ceased at least 3 months prior to the recruitment, the effect of high levels of vitamin D on Th17 and Treg frequency may exist. However, we could not comment on its effect, as not all the women were on vitamin D supplementation and we did not have any records on the duration and dosage, Nevertheless, observations from our study may pave a path for future studies focusing on the additional factors apart from estrogen insufficiency that are responsible for inflammation which results in low BMD.

Conclusively, estrogen insufficiency post menopause leads to elevated circulating Th17 frequency that alters Th17/Treg balance, causing inflammation in the bone microenvironment. Th17 cells play an important role in the pathogenesis of osteoporosis and higher Th17 cell frequency may be a distinctive marker for the diagnosis of osteoporosis, which should be validated in the larger study samples. Furthermore, suppressing critical cytokines like IL-17 may aid in the development of a potential immunotherapeutic approach for the treatment of osteoporosis.

## Methods

### Study subjects

The study participants were selected by the Clinicians after screening Dual energy X-ray Absorptiometry (DXA, Lunar iDXA, GE Healthcare, USA) scans of 620 women available from two different centers. Women with a history of smoking, alcohol consumption, or chronic disorders, endocrinopathy, diabetes, and hypertension, pregnant, lactating, on oral contraceptives, glucocorticoids, hormone replacement therapy; drugs affecting bone mass and immune system were excluded from the study. Women with no acute illness, physically active, non-smokers, non-consumer of alcohol and normotensive were included in the study. Finally, 255 healthy participants were selected for the study, of which 140 premenopausal women were in the age group of 25–40 years and 115 postmenopausal women were in the age group of 50–65 years. All premenopausal women had regular menstrual cycles for the last 6 months and postmenopausal women had attained natural menopause at least a year prior to enrolment. Written informed consent was obtained from each participant and their clinical history was recorded. All the subjects were from the city of Mumbai, India and the study was reviewed and approved by the ICMR-NIRRH Institutional Ethical Committee for Clinical Studies (Project ID 259/2014). We further confirm that all the research was performed in accordance with the relevant guidelines and regulations of the committee.

Initially, All selected postmenopausal women were classified according to WHO criteria as normal (T score ≥ − 1.0 at both the sites), osteopenic (T score between − 1.0 and − 2.5 at any site) and osteoporotic (T score ≤ − 2.5 at any site). However, for our study, all the enrolled postmenopausal women were either having normal BMD (Post-N) or had osteoporosis (Post-L). Premenopausal women enrolled in the study had normal BMD (Pre-N). A total of 70 women (Pre-N: n = 25, Post-N: n = 20, and Post-L: n = 25) who were willing to participate were enrolled for the study.

Fasting blood 10 mL was drawn from each participant between 9 and 10 a.m. into EDTA (BD Biosciences, San Jose, USA) and plain tubes (BD Biosciences, San Jose, USA). Blood sample of premenopausal women was drawn on any day between days 12–14 of their menstrual cycle, whereas, for postmenopausal women, the sample was drawn on any random day. A part of whole blood was used for immunophenotyping while the remaining whole blood was used for separation of peripheral blood mononuclear cells (PBMC) and plasma. The plasma samples were stored at − 80 °C in aliquots till further use. PBMCs were immediately used for flow cytometry analysis and a part of it was cryopreserved for future analysis. The estimations of cytokines and hormones were done in plasma samples whereas calcium and phosphorous were estimated from serum samples.

### Immunophenotypic analysis of T cell subsets

Whole blood (200 μl) was stained with fluorochrome-conjugated antibodies. The panel used for the cellular surface markers consisted of CD3 (clone: SK7), CD4 (clone: RPA-T4), CD25 (clone: MA251) and CD127 (clone: HIL-7R-M21) (Biolegend, San Diego, USA and BD Biosciences, San Jose, USA). After staining for 30 min in dark at room temperature, cells were fixed with BD FACS lysing solution followed by red blood cells lysis in accordance with the manufacturer’s protocol (BD Biosciences, San Jose, USA). Lastly, cells were washed at 350 g for 10 min and resuspended in PBS with 0.2% BSA. Acquisition and analysis were performed on 1 million acquired events on BD accuri C6 flow cytometer equipped with the 4 lasers (BD Biosciences, San Jose, USA). The fluorescent minus one (FMO) gating strategy was applied to identify positive cells to discriminate the background autofluorescence and negative population. Data was processed and analyzed using FlowJo 10 (Tree Star Inc., Ashland, USA).

### Intracellular cytokine staining assay

PBMCs were isolated from whole blood by density gradient centrifugation using Histopaque 1077 (Sigma Aldrich, St Louis, USA) followed by its stimulation with 50 ng/ml phorbol-12-myristate-13-acetate (PMA) and 1 µg/ml ionomycin (Sigma Aldrich, St Louis, USA) in the presence of Brefeldin A (BD Biosciences, San Jose, USA) for 6 h at 37 °C in a 5% CO_2_ incubator. Following incubation, cells were washed and surface stained by incubating it (1–2 × 10^6^ cells/ml) with anti-human monoclonal antibodies (anti-CD3-FITC, clone: SK7 and CD8-PeCy7, clone: SK1) (Biolegend, San Diego, USA). Cells were then fixed and permeabilized using BD cytofix/cytoperm kit (BD Biosciences, San Jose, USA) according to the manufacturer’s protocol. The permeabilized cells were stained for intracellular IL-17 (clone: SCPL1362) and IL-10 (clone: JES3-9D7) at 4 °C for 30 min. After staining, the cell pellet was washed and resuspended in stain buffer. 1 million events were acquired using BD accuri C6 flow cytometer (BD Biosciences, San Jose, USA). The analysis was performed using FlowJo 10 (Tree Star Inc., Ashland, USA).

### Hormone and cytokine estimations

Plasma levels of Estradiol (DBC, Canada), 25(OH) Vitamin D (Calbiotech, Cajon, USA), C-Reactive protein (CRP) (Calbiotech, Cajon, USA) and Transforming growth factor-β (TGF-β) (ThermoFisher Scientific, Waltham, USA) were estimated using commercially available ELISA kits as per the manufacturer's protocol. The intra and inter-assay coefficients of variation for all the kits were < 8% and < 12% respectively. The concentrations were determined from standard curves using Gen5.0 (BioTek, USA). Serum levels of calcium and phosphorous were determined using an autoanalyzer (ERBA, Manheim, Germany).

Plasma levels of IL-17A, IL-6, TNF-α, IL-10, IL-4, IFN-γ and IL-7 were estimated simultaneously from a single aliquot of each sample using human ProcartaPlex multiplex immunoassay (ThermoFisher Scientific, Waltham, USA) and quantified using Luminex 200 detection system equipped with Xponent 4.2. The lower detection limits for each cytokine were 0.1 pg/ml (IL-17A), 0.21 pg/ml (IL-6), 0.4 pg/ml (TNF-α), 0.2 pg/ml (IFN-γ), 0.2 pg/ml (IL-7), 0.1 pg/ml (IL-10) and 0.03 pg/ml (IL-4).

### Statistical analysis

Statistical analysis was carried out using SPSS Statistics for Windows, Version 16.0 (SPSS Inc., Chicago, III., USA) and GraphPad Prism 5.0 (GraphPad Software, Inc., San Diego, USA). Multiple group comparison was done using the Kruskal–Wallis test with Dunn’s post-hoc analysis and pairwise comparison was done using Mann–Whitney U-test. Non-parametric Spearman’s rank correlation was used to assess the correlation between the two variables. Differences between groups were considered statistically significant when the *p-value* was < 0.05.

## Supplementary Information

Supplementary Information.

## Data Availability

The datasets generated for this study are available on request to the corresponding author.
