# Supplementary material for: Increased frequency of Th17 cells and IL-17 levels are associated with low bone mineral density in postmenopausal women
Source: Sci Rep. 2021 Aug 9;11:16155. doi: 10.1038/s41598-021-95640-0 (PMC8352954; doi:10.1038/s41598-021-95640-0)
Supplement: Supplementary file 1 — Supplementary Information. [file 41598_2021_95640_MOESM1_ESM.docx]

**Supplementary Material**

**Supplementary Figure 1:**

**
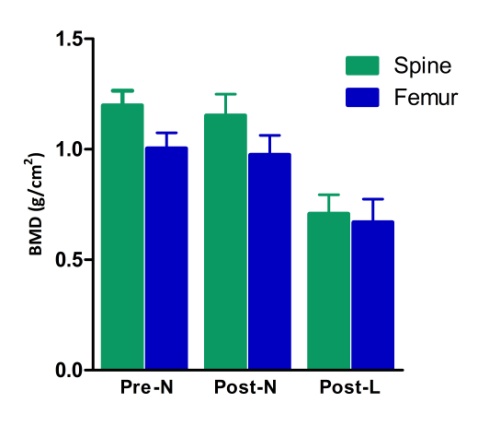
**

**Suppl. Fig. 1:** Comparison of BMD measurement between premenopausal women with normal BMD (Pre-N), postmenopausal women with normal BMD (Post-N) and postmenopausal women with low BMD (Post-L).

**Supplementary Figure 2:**

**
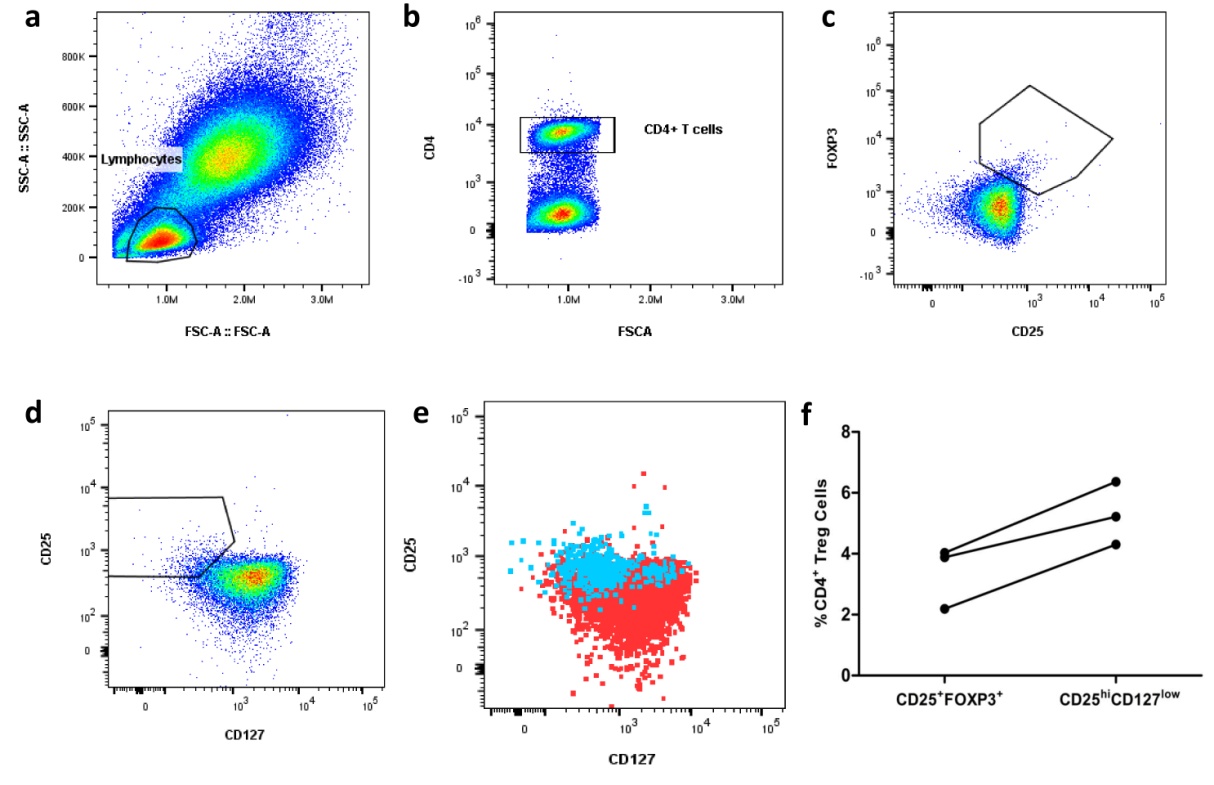
**

**Suppl. Fig. 2: Representative plot of validation of CD4^+^CD25^hi^CD127^-/low^ phenotype as Tregs of 3 healthy samples:**

Based on forward and side scatter lymphocyte population was gated **(a)**, followed by gating of CD4^+^ T cells **(b)**. Thereafter CD4^+^ T lymphocytes were analyzed for CD25 and FOXP3 expression **(c),** and CD25 and CD127 expression **(d)**. Light blue dots indicate CD25^hi^CD127^low/-^ population, which largely overlaps with CD25^+^FOXP3^+^ T cells, which was obtained by overlaying plot **d on plot c of** CD4+CD25+FOXP3+ **(e).** The frequencies of the CD4^+^CD25^+^FOXP3^+^ and CD4+CD25^high^CD127^low^ population were obtained through the concurrent staining from 3 healthy individuals **(f).**

**Supplementary Figure 3:**


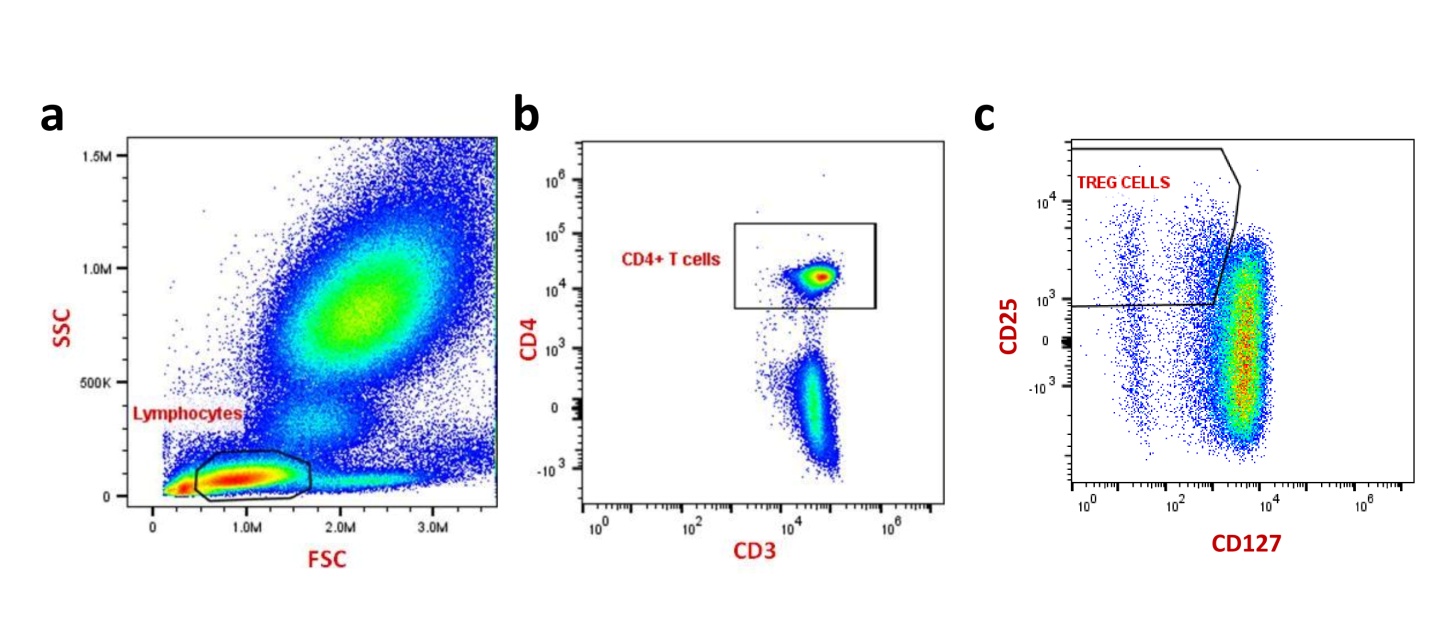


**Suppl. Fig. 3: Gating strategy to categorize Regulatory T cells (Treg) using CD25 and CD127 markers.** A lymphocyte Population (a) was gated followed by gates set on CD3^+^CD4^+^ for CD25 and CD127 positivity (b). Based on CD25 and CD127 expression within CD4 compartment, population was further marked as Treg (CD3^+^CD4^+^CD25^hi^ CD127^low/-^) (c).

**Supplementary Figure 4:**


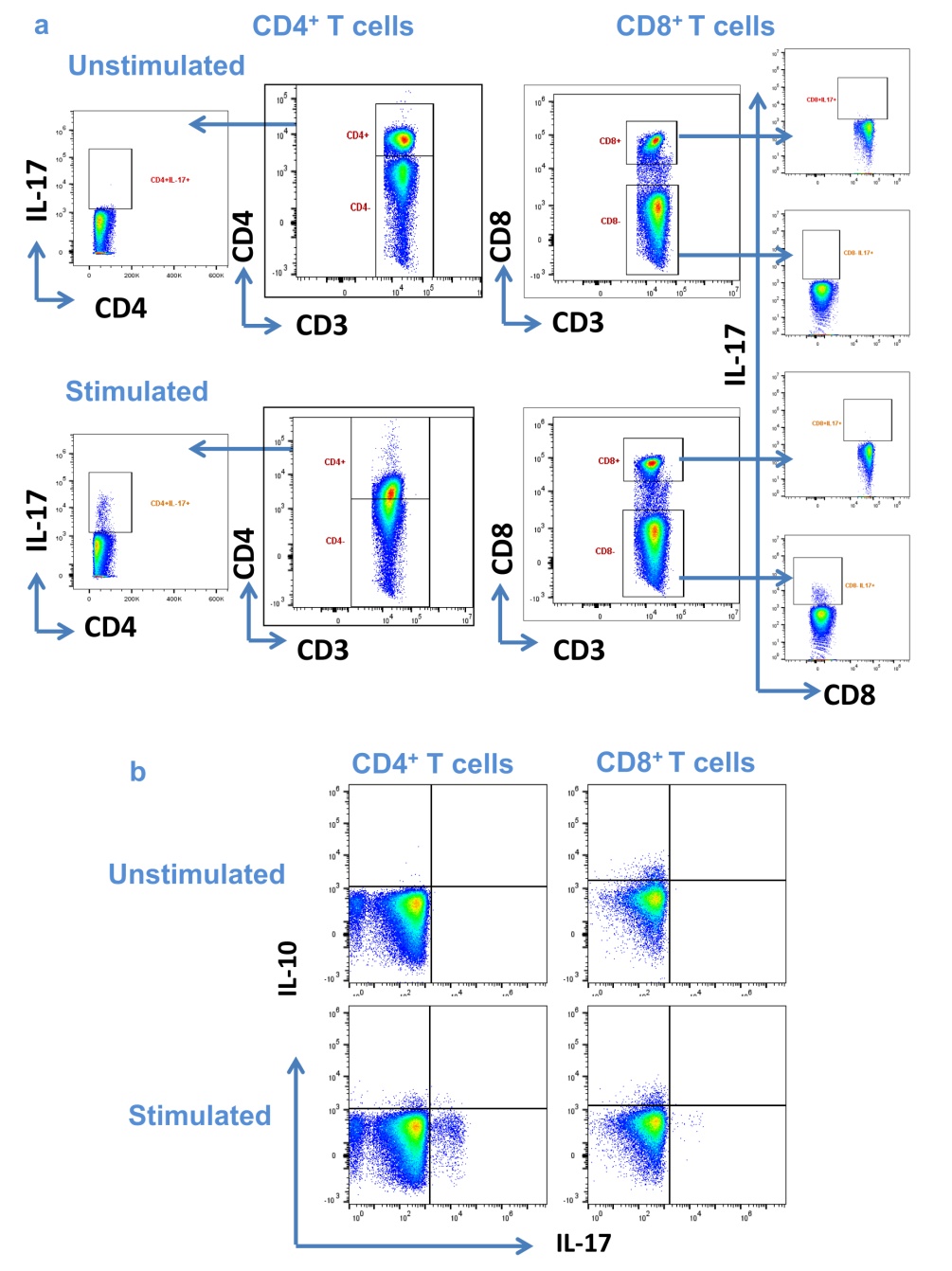


**Suppl. Fig. 4: Gating strategy to identify Th17 using CD4 and CD8 markers in PBMC stimulated with PMA/Ionomycin.** (a) Initially, lymphocytes were gated based on forward and side scatter properties of light followed by gating of CD4^+^ and CD8^+^ T cells. Further, based on IL-17 expression, CD4^+^ and CD8^+^ T cells were defined as IL-17^+^ CD4^+^ or IL-17^+^ CD8^+^ (compared to unstimulated) population.

Gating strategy to identify Th17 and IL10^+^ T cells (b). Initially, lymphocytes were gated based on forward and side scatter properties of light followed by gating of CD8^-^ and CD8^+^ T cells based on the expression of CD3 and CD8 markers. Further, based on IL-17 and IL-10 expression, CD4^+^ and CD8^+^ T cells were defined as Th17 CD8^-^ IL-17^+^, CD8^-^ IL-10^+^ and CD8^+^ IL-10^+^ populations.

**Supplementary Table 1: Mean plasma levels of cytokine in women with normal and low BMD**

| Cytokines  (pg/ml) | Pre-N  (n = 21) | Post-N  (n = 20 ) | Post-L  (n = 20) |
| --- | --- | --- | --- |
| IFN-γ | 3.71 ± 0.71 | 4.10 ± 1.1 | **4.35** ± **1.07*** |
| IL-7 | 1.98 ± 1.28 | **3.08** ± **2.3** | 2.60 ± 2.09 |
| IL-4 | 2.56 ± 1.84 | **3.77** ± **2.54*** | **4.2** ± **2.38*** |

**Suppl. Table 1. Plasma cytokines level in premenopausal and postmenopausal with normal and low BMD.** Plasma cytokine levels were estimated by Luminex immunoassay. Values are expressed as mean ± SD. **p* < 0.05; ***p* < 0.01 and ****p* < 0.0001 when compared to Pre-N. Statistical significance was estimated using the Mann-Whitney U test. Pre-N, premenopausal women with normal BMD, Post-N, postmenopausal women with normal BMD, Post-L, postmenopausal women with low BMD.
